# Therapy for acute basilar artery occlusion: a systematic review and meta-analysis

**DOI:** 10.12688/f1000research.18042.1

**Published:** 2019-02-07

**Authors:** Kevin Sheng, Marcus Tong

**Affiliations:** 1 Faculty of Medicine, Macquarie University, Macquarie Park, NSW, 2109, Australia; 2 Concord Hospital, Concord, NSW, 2137, Australia; 3 Sir Charles Gairdner Hospital, Nedlands, Nedlands, WA, 6009, Australia

**Keywords:** basilar, occlusion, thrombolysis, thrombectomy, aspiration, stent retreiver, thromboaspiration, intraarterial

## Abstract

**Purpose:** This study aims to analyse the efficacy of different treatment methods for acute basilar artery occlusion, with an emphasis placed on evaluating the latest treatment methods.

**Method:**  A systematic review and meta-analysis was performed to analyse the current data on the therapies available for treating acute basilar artery occlusion.

**Results: **A total of 102 articles were included. The weighted pooled rate of mortality was 43.16% (95% CI 38.35-48.03%) in the intravenous thrombolysis group, 45.56% (95% CI 39.88-51.28) in the intra-arterial thrombolysis group, and 31.40% (95% CI 28.31-34.56%) for the endovascular thrombectomy group. The weighted pooled rate of Modified Ranking Score (mRS) 0-2 at 3 months was 31.40 (95% CI 28.31-34.56%) in the IVT group, 28.29% (95% CI 23.16-33.69%) in the IAT group, and 35.22% (95% CI 32.39-38.09%) for the EVT group. Meta-analyses were also done for the secondary outcomes of recanalization and symptomatic haemorrhage. There was no difference between stent retriever and thrombo-aspiration thrombectomy on subgroup analysis in both clinical outcome and safety profile.

**Limitations**: The included studies were observational in nature. There was significant heterogeneity in some of the outcomes.

**Conclusions:**  Superior outcomes and better recanalization rates for acute basilar occlusion were seen with patients managed with endovascular thrombectomy when compared with either intravenous and/or intraarterial thrombolysis. No superiority of stent‐retrievers over thrombo-aspiration thrombectomy was seen.

## Introduction

Strokes caused by basilar artery occlusion are uncommon, with around 10% of large vessel strokes being basilar ^1^. They are associated with very poor outcomes and high mortality; however, the condition can be heterogenous with variable prognosis ^2^. There are a number of different pathophysiological mechanisms, including atherosclerosis, embolism, dissection and inflammation ^3^. The presentation is variable, with patients presenting in different ways. In the preceding two to three weeks, many people may suffer from a prodrome that includes symptoms such as headache and vertigo ^4^. On admission, patients may present with decreased GCS, dysarthria, focal weakness or cranial nerve dysfunction, ataxia and abnormal pupillary signs.

Early and successful recanalization has been shown to result in better outcomes in patients treated with intravenous thrombolysis and endovascular therapy ^5^. While intravenous thrombolysis remains first line therapy for patients who present within the time-frame, previous studies show a low recanalization rate and poor clinical outcomes ^6, 7^. In strokes of the proximal anterior circulation, early mechanical thrombectomy has been shown through multiple randomised controlled trials to be superior to intravenous thrombolysis alone ^8– 10^. To date, there has only been a single small randomised clinical trial of 16 patients that compared intra-arterial urokinase versus control, however the study was prematurely stopped due to poor recruitment ^11^. There are currently no other randomised trial data comparing different therapeutic approaches for basilar artery occlusion, with only observational data available.

Observational data has suggested higher recanalization rates and better clinical outcomes for patients treated with endovascular thrombectomy by stent retrievers and thrombo-aspiration devices, and new techniques such as a direct-aspiration first-pass technique (ADAPT) and Combined Stent Retriever and Suction Thrombectomy (Solumbra technique) are promising. Hence it was decided to perform a systematic review and meta-analysis of the different therapeutic interventions for basilar artery occlusion, with a focus on comparing the safety profile and clinical performance of stent retriever vs thrombo-aspiration thrombectomy.

### Natural history of disease

Data from the New England Medical Centre Posterior Circulation Registry shows that most of the patients with a basilar artery occlusion are between the ages of 50 and 80 ^2, 3^. Important risk factors for this disease include hypertension, diabetes mellitus, hypertension, hyperlipidaemia, smoking status, peripheral arterial disease and prior stroke. A large proportion of patients have been shown to have mini-strokes prior to the onset of basilar artery occlusion, with 58.6% of those with transient ischemic attack proceeding onto stroke.

When managed conservatively, the literature presents a mixed but grim picture regarding prognosis. Most studies show poor outcomes of death and dependency in up to 95% of the study population ^12– 14^, however two observational studies show favourable outcomes in 71% (n=87) and 71% (n=61) respectively of patients managed conservatively ^3, 15^. This is likely reflective of numerous factors including study factors such as patient eligibility criteria and definition of outcomes. However, it also reflects the heterogenous nature of basilar artery occlusion, which has a variable severity and prognosis but can often be fatal ^2^.

Voetsch *et al.* analysed a group of 87 patients with moderate to severe basilar artery stenosis or occlusion from the New England Medical Centre Posterior Circulation Registry ^3^. From this study, there were several factors that were statistically significantly associated with worse outcome. These included most significantly distal territory involvement and occlusion secondary to embolism, which likely reflects the lack of time for a collateral blood supply to form. Clinical predictors of worse clinical outcomes included decreased GCS on presentation, tetraplegia and pupillary signs. These predictors are supported by analysis of patients from the Lausanne Registry, which shows decreased GCS was the most important clinical predictor of poor outcome ^16^.

## Method

### Search strategy

PubMed was searched (through to August 2018) to identify pertinent research articles with the keyword “basilar”. The search strategy was deliberately kept simple in order to prevent the omission of large numbers of studies that may have occurred with a more restrictive search strategy.

The only limitations applied included article types (clinical study, clinical trial, comparative study, controlled clinical trial, dataset, evaluation studies, journal article, multicentre study, observational study, randomised controlled trial, twin study, validation studies), species (humans) and ages (Adult: 19+ years). Titles and abstracts were reviewed, with articles accessed in their entirety if they were potentially appropriate. Reference lists of research articles were further parsed for additional potentially appropriate studies. Research articles were read in their entirety if a decision on study inclusion could not be determined by reading the abstract. Attempts were made to communicate with the corresponding authors if further information was required. 

All studies regardless of project design (including retrospective and prospective) were permitted. The limitation to this was that studies needed to have at a minimum of 10 patients, with studies reporting less than this being excluded. Studies were included if they included treatment for acute basilar artery occlusion. Observational and interventional studies covering intravenous thrombolytic therapy (IVT), intra-arterial thrombolytic therapy (IAT), plus/minus endovascular therapy (EVT) were allowed.

Other exclusion criteria not already mentioned included the following: full text unavailable, duplicate studies, intervention other than IVT/IAT/EVT, lack of information regarding primary and secondary study outcomes (mortality, MRS 0-2, symptomatic intracranial haemorrhage, recanalization).

### Data extraction

Data was independently parsed into a standardised table on Microsoft Excel 2013 by the two authors (KS and MT). Relevant data abstracted included method of therapy (IVT/IAT/EVT), clinical outcome at 3 months (Modified Rankin Score, Barthel Index, other), study population, baseline NIHSS, age, mortality, intracranial haemorrhage, study design, recanalization status, country, data collection window, time to first groin puncture, complications (dissection, perforation, embolization to new territory), adjunctive therapy. Data was combined into a single table once this process was complete.

Primary outcomes were mortality and good clinical outcome (mRS 0-2). Secondary outcomes were symptomatic intracranial haemorrhage (SICH) and recanalization. For the analysis comparing the stent retriever and thrombo-aspiration subgroups, the additional outcomes of dissection/perforation and embolization to new territory were parsed.

Mortality was assessed at 90 days, however if this was not available, then the nearest value was imputed as a surrogate. Clinical outcome was determined to be good if the mRS score was 0-2. If another definition was provided by the study and there was no other information to calculate the mRS score, then that particular definition was used. Clinical outcome was ideally determined at 3 months, but if this was unavailable or not determined, then the nearest value was imputed as a surrogate.

SICH was defined as any haemorrhage associated with a worsening of the NIHSS score by ≥4 within 24 h, in accordance with the ECASS-II definition ^17^. If another definition was provided by the study, then that particular definition was used. Recanalization was defined as TICI 2b/3, mTICI 2b/3, TIMI 2/3, or as per the study definition.

Differences were resolved through discussion and consensus of the two reviewers. Quality of studies was determined for each paper according to the reporting checklist proposed by the Meta-analysis of Observational Studies in Epidemiology (MOOSE) group.

### Statistical analysis

***Data synthesis.*** The DerSimonian and Laird random effects model was applied to perform the meta-analysis. If there was no significant heterogeneity, then the fixed effect model was used. In order to generate standard errors, the Freeman-Tukey double arc-sine transformation was applied to data extracted from individual studies. These are then back-transformed in order to form mean weighted probabilities with 95% confidence intervals.

***Assessment of heterogeneity and publication bias.*** Heterogeneity was evaluated through Cochran’s χ2 test (Cochran Q test), tau-squared and Higgin’s I-square statistic. A p-value of less than 0.05 and I-square >50% was regarded as significant. Publication bias was assessed through a variety of methods. These included the Begg and Mazumdar’s rank correlation test and Egger’s linear regression test (with a significance of p<0.05). In addition, funnel plots were generated, and trim and fill plot analysis was also conducted to adjust for any significant publication risk and publication bias adjusted weighted pooled rates are calculated.

***Sensitivity analysis.*** Subgroup analyses and meta-regression were performed to determine potential sources of heterogeneity. The subgroups included intra-arterial thrombolysis (with angioplasty +/- stenting), intra-arterial thrombolysis (without angioplasty +/- stenting), stent retriever thrombectomy and thrombo-aspiration thrombectomy. For the stent retriever and thrombo-aspiration subgroups, studies were only included if data could be parsed specifically for outcomes relating to each. Meta-regression was done to compare outcome against publication year and time to first puncture. Comparison of subgroups was undertaken using the z-test of interaction. Exclusion sensitivity analysis was also performed.

***Software.*** All analyses and calculations in this meta-analysis were performed using the Mix V2.0 Pro statistical package.

## Results

### Search results

**Figure 1.**
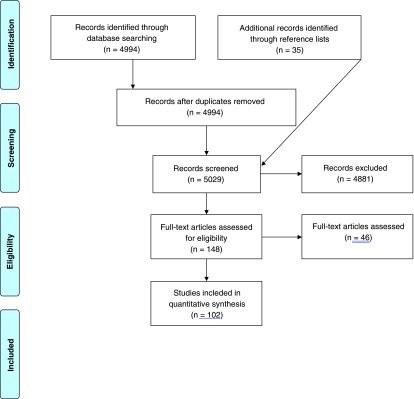
PRISMA flow diagram.

A total of 4994 articles were identified from PubMed. After evaluating the titles and abstracts of these articles, 148 remained eligible for assessment. The full texts of these articles were assessed, and 102 articles fulfilled the inclusion criteria. A PRISMA flow diagram has been included ( Figure 1).

### Primary outcomes for the IVT, IAT and EVT groups

**Figure 2.**
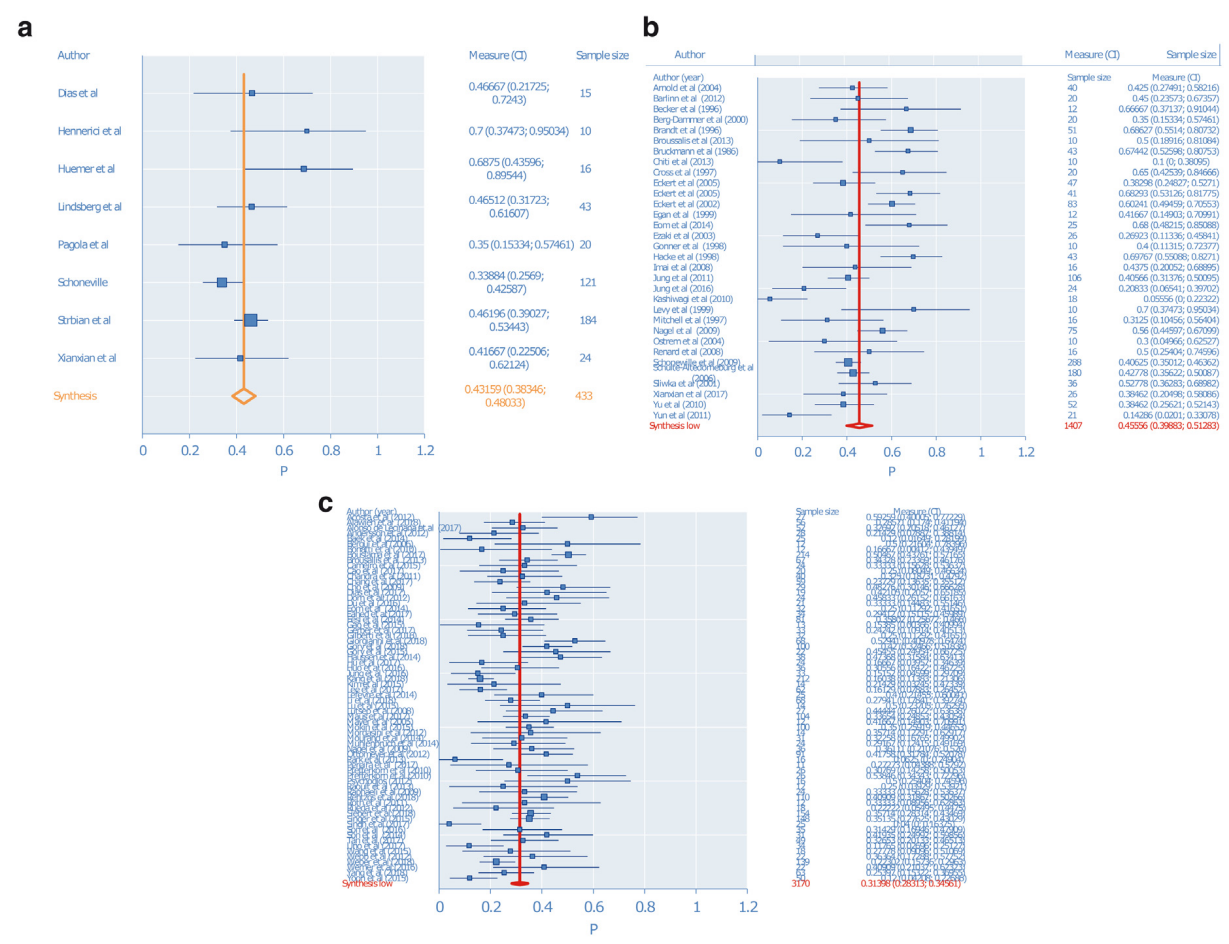
Meta-analysis for mortality in the ( **a**) intravenous thrombolysis subgroup; ( **b**) intra-arterial thrombolysis subgroup; ( **c**) endovascular thrombectomy subgroup.

**Figure 3.**
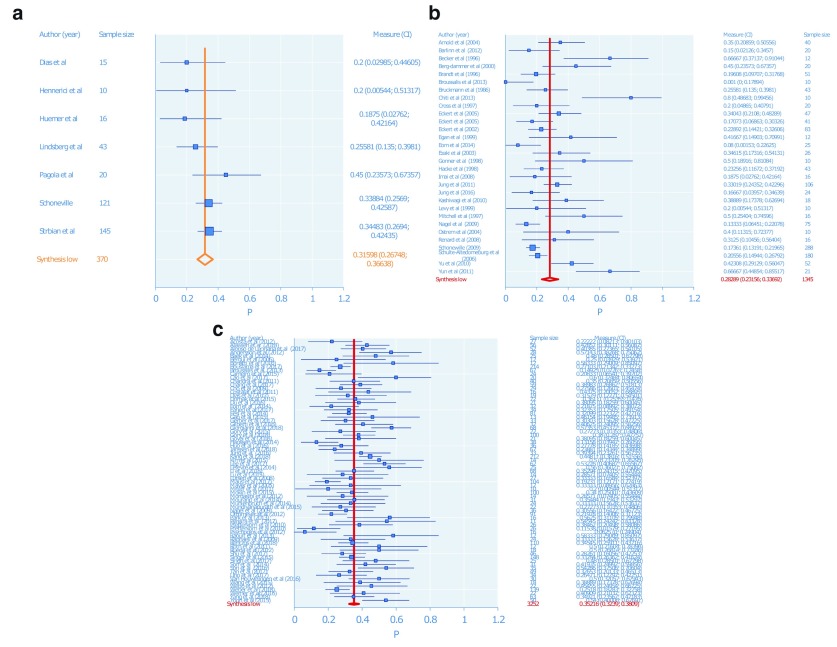
Meta-analysis for good outcome in the ( **a**) intravenous thrombolysis subgroup; ( **b**) intraarterial thrombolysis subgroup; ( **c**) endovascular thrombectomy subgroup.

See Table 1 and Table 2 and Figure 2a–c and Figure 3.

**Table 1.** Heterogeneity and mean weighted probabilities for mortality. IVT, Intravenous thrombolysis. IAT, Intraarterial thrombolysis. EVT, Endovascular thrombolysis. SR, Stent retriever. TA, Thromboaspiration. MWP, mean weighted probability. CI, confidence interval. Q, Cochran’s Q. AP, angioplasty.

Outcome of Interest	MWP	CI	Begg’s, Egger’s test	Trim and fill adjusted MWP	Q/p	I2	T2	N
Mortality IVT	43.16%	38.35% to 48.03%, p<0.0001	0.54, 0.24	N/a	15.24, 0.055	44.527	0.00443	433
Mortality IAT	45.56%	39.88% to 51.28%, p<0.0001	0.52, 0.87	46.55%, 43.86% to 49.25%	112.44, 0.00	72.43	0.016	1407
Mortality IAT (no AP or stenting)	49.79%	43.47% to 56.11%, p<0.0001	-	-	59.69, 0.00001	64.82	0.011	989
Mortality IAT + AP/stent	36.55%	25.16% to 48.68%, p<0.0001	-	-	49.09, 0	81.67	0.028	418
Mortality EVT	31.40%	28.31% to 34.56%, p<0.0001	0.56, 0.63	N/a	202.53	66.92	0.011	3170
Mortality SR EVT	24.83%	22.03% to 27.74%, p<0.0001	0.57, 0.034	N/a	57.54, 0.00124	49.60	0.0077	967
Mortality TA EVT	25.97%	17.57% to 35.28%, p<0.0001	0.53, 0.67	24.07%, 19.42% to 29.02%	25.62, 0.0024	64.87	0.015	317

**Table 2.** Heterogeneity and mean weighted probabilities for good outcome. IVT, Intravenous thrombolysis. IAT, Intraarterial thrombolysis. EVT, Endovascular thrombolysis. SR, Stent retriever. TA, Thromboaspiration. MWP, mean weighted probability. CI, confidence interval. Q, Cochran’s Q. AP, angioplasty.

Outcome of Interest	MWP	CI	Begg’s, Egger’s test	Trim and fill	Q/p	I2	T2	N
Good outcome IVT	31.60%	26.75% to 36.64%, p<0.0001	0.55, 0.15	N/a	5.33, 0.50	0.00	0.00	370
Good outcome IAT	28.29%	23.16% to 33.69%, p<0.0001	0.093, 0.0036	21.71%, 19.50% to 24.01%	101.26, 0.00	71.36	0.015	1345
Good outcome IAT (no AP or stenting)	26.48%	20.29% to 33.11%, p<0.0001	-	-	64.30, 0.00	70.45	0.014	927
Good outcome IAT (with AP or stenting)	31.50%	23.49% to 40.06%, p<0.0001	-	-	25.21, 0.0028	64.30	0.011	418
Good outcome EVT	35.22%	32.39% to 38.09%, p<0.0001	0.095, 0.10	N/a	175.45, 0.00	58.96	0.0081	3252
Good outcome SR EVT	39.02%	35.74% to 42.35%, p<0.0001	0.56, 0.98	N/a	42.65, 0.079	27.32	0.0033	903
Good outcome TA EVT	35.45%	30.52% to 40.52%, p<0.0001	0.73, 0.66	N/a	20.35, 0.041	45.96	0.0068	377

### Secondary outcomes for the IVT, IAT and EVT groups

See Table 3 and Table 4.

**Table 3.** Heterogeneity and mean weighted probabilities for recanalization. IVT, Intravenous thrombolysis. IAT, Intraarterial thrombolysis. EVT, Endovascular thrombolysis. SR, Stent retriever. TA, Thromboaspiration. MWP, mean weighted probability. CI, confidence interval. Q, Cochran’s Q. AP, angioplasty.

Outcome of Interest	MWP	CI	Begg’s, Egger’s test	Trim and fill	Q/p	I2	T2	N
Recanalization IVT	63.77%	58.64% to 68.76% p<0.0001	0.089, 0.11	67.13%, 62.43% to 71.68%	11.41, 0.12	38.65	0.0039	380
Recanalization IA	71.79%	66.29% to 77.01%, p<0.0001	0.25, 0.53	69.70%, 67.16 to 72.18%	113.21, 0.00	73.5	0.016	1381
Recanalization IA (no AP or stenting)	71.54%	63.91% to 78.64%, p<0.0001	-	-	94.05, 0.00	78.74	0.022	963
Recanalization AP (with AP or stenting)	72.38%	65.17% to 79.09%, p<0.0001	-	-	19.01, 0.025	52.65	0.0070	418
Recanalization EVT	84.48%	81.82% to 86.97%, p<0.0001	0.65, 0.90	N/a	278.95, 0.00	72.76	0.015	3544
Recanalization SR EVT	87.22%	83.48% to 90.59%, p<0.0001	0.56, 0.81	N/a	85.45, 0	60.21	0.012	1119
Recanalization TA EVT	92.99%	88.03% to 96.87%, p<0.0001	0.93, 0.69	N/a	18.33, 0.032	50.91	0.0076	345

**Table 4.** Heterogeneity and mean weighted probabilities for recanalization. IVT, Intravenous thrombolysis. IAT, Intraarterial thrombolysis. EVT, Endovascular thrombolysis. SR, Stent retriever. TA, Thromboaspiration. MWP, mean weighted probability. CI, confidence interval. Q, Cochran’s Q. AP, angioplasty.

Outcome of Interest	MWP	CI	Begg’s, Egger’s test	Trim and fill	Q/p	I2	T2	N
Symptomatic ICH IVT	8.52%	3.80% to 14.54%, p<0.0001	1.00, 0.25	15.20%, 12.19% to 18.44%	13.52, 0.036	55.61	0.0064	417
Symptomatic ICH IA	8.91%	7.22% to 10.73%, p<0.0001	0.55, 0.74	8.72%, 7.05% to 10.52%	42.79, 0.027	36.90	0.0034	1267
Symptomatic ICH IA (no AP or stenting)	10.22%	8.04% to 12.9%, p<0.0001	-	-	17.89, 0.40	4.96	0.00031	849
Symptomatic ICH IA (with AP or stenting)	8.05%	3.99% to 13.11%, p<0.0001	-	-	19.61, 0.020	54.12	0.0074	418
Symptomatic ICH EVT	5.39%	4.43% to 6.40%, p<0.0001	0.31, 0.034	3.51%, 2.78% to 4.29%	106.08, 0.00012	45.32	0.0047	2637
Symptomatic ICH SR EVT	5.13%	3.45% to 7.05%, p<0.0001	0.020, 0.10	2.72%, 1.58% to 4.07%	28.19, 0.40	4.22	0.00039	787
Symptomatic ICH TA EVT	2.93%	0.76% to 6.00%, p<0.0001	0.27, 0.28	N/a	5.53, 0.60	0.00	0.00	343

### Z-test of interaction subgroup analysis

See Table 5 and Table 6. The z test of interaction between the weighted pool rate for mortality in the EVT group versus the IAT and IVT was statistically significant, indicating that the treatment effect likely differs between the groups.

**Table 5.** Mortality subgroup comparison. IVT, Intravenous thrombolysis. IAT, Intraarterial thrombolysis. EVT, Endovascular thrombolysis.

Comparison	z	p
EVT vs IAT	3.17225	0.00151
EVT vs IVT	2.65194	0.008

**Table 6.** Good outcome subgroup comparison. IVT, Intravenous thrombolysis. IAT, Intraarterial thrombolysis. EVT, Endovascular thrombolysis.

Comparison	z	p
EVT vs IAT	2.24286	0.02491
EVT vs IVT	0.91977	0.35769

The z test of interaction between the weighted pool rate for good clinical outcome in the EVT group versus IVT was not statistically significant but was statistically significant compared to the IAT group.

### Subgroup analysis (stent retriever vs thrombo-aspiration)

See Table 7. The z test of interaction between the weighted pool rates for the above clinical outcomes in the stent retriever (SR) versus thromboaspiration (TA) groups was not statistically significant, indicating that the treatment effect likely did not differ between the groups.

**Table 7.** Heterogeneity and mean weighted probabilities for Dissection + Perforation and Embolization to new territory. EVT, Endovascular thrombolysis. SR, Stent retriever. TA, Thromboaspiration. MWP, mean weighted probability. CI, confidence interval. Q, Cochran’s Q.

Outcome of Interest	MWP	CI	Begg’s, Egger’s test	Trim and fill	Q/p	I2	T2	N
Dissection + Perforation SR EVT	3.71%	1.88% to 5.96%, p<0.0001	0.064, 0.12	N/a	12.63 0.86	0.00	0.00	484
Dissection + Perforation TA EVT	2.49%	0.54% to 5.34%, p<0.0001	0.14, 0.43	2.36%, 0.50% to 5.09%	1.37, 0.99	0.00	0.00	250
Dissection + Perforation EVT	3.67%	2.63% to 4.84%, p<0.0001	0.13, 0.03	N/a	29.92, 0.79	0.00	0.00	1583
Embolization to new territory SR EVT	17.63%	12.71% to 23.07%, p<0.0001	0.75, 0.57	N/a	10.89, 0.89	26.56	0.0035	237
Embolization to new territory TA EVT	13.18%	1.29% to 32.22%, p=0.012	0.46, 0.060	N/a	24.14, 0.00007	83.43	0.050	130
Embolization to new territory EVT	15.41%	8.89% to 23.11%, p<0.0001	0.081, 0.054	N/a	117.78, 0.00	85.57	0.030	930

### Meta-regression

See Table 8 and Table 9.

**Table 8.** Meta-regression of publication year against primary and secondary. Estimate, slope co-efficient. se, standard error. ci-/ci+, confidence interval. z, z value.

Event (covariate)	estimate	se	ci-	ci+	z	p
IVT mortality (publication year)	-0.00813	0.00388	-0.01573	-0.00053	-2.09751	0.03595
IAT mortality (publication year)	-0.01293	0.0022	-0.01724	-0.00862	-5.87672	<0.001
EVT mortality (publication year)	-0.01202	0.00341	-0.01869	-0.00535	-3.53013	<0.001
IVT mRS 0-2 (publication year)	0.00543	0.00474	-0.00386	0.01473	1.14623	0.2517
IAT mRS 0-2 (publication year)	-0.00353	0.00229	-0.00802	0.00097	-1.53635	0.12445
EVT mRS 0-2 (publication year)	0.00652	0.00331	0.00004	0.01299	1.97177	0.04864
IVT recanalization (publication year)	0.00479	0.00398	-0.00302	0.0126	1.20211	0.22932
IAT recanalization (publication year)	-0.00555	0.00231	-0.01008	-0.00103	-2.40567	0.01614
EVT recanalization (publication year)	0.00652	0.00331	0.00004	0.01299	1.97177	0.04864
IVT SICH (publication year)	0.00581	0.00459	-0.00318	0.0148	1.26763	0.20493
IAT SICH (publication year)	0.00148	0.00232	-0.00306	0.00603	0.64004	0.52215
EVT SICH (publication year)	-0.02111	0.00338	-0.02773	-0.0145	-6.25381	<0.001

**Table 9.** Meta-regression of time to groin puncture against primary and secondary. Estimate, slope co-efficient. se, standard error. ci-/ci+, confidence interval. z, z value.

Event (covariate)	estimate	se	ci-	ci+	z	p
IAT mortality (time to groin puncture)	0.0002	0.00008	0.00005	0.00035	2.64396	0.00819
EVT mortality (time to groin puncture)	0.00037	0.00006	0.00026	0.00048	6.55891	<0.001
IAT good clinical outcome (time to groin puncture)	-0.00001	0.00008	-0.00016	0.00015	-0.08626	0.93126
EVT good clinical outcome (time to groin puncture)	-0.00016	0.00005	-0.00027	-0.00005	-2.90737	0.00364
IAT recanalization (time to groin puncture)	0.00002	0.00008	-0.00014	0.00018	0.28285	0.77729
EVT recanalization (publication year)	0.00002	0.00006	-0.00009	0.00013	0.38155	0.7028

Meta-regression demonstrated a statistically significant correlation towards decreased mortality across time with IVT, IAT and EVT therapy. There was a statistically significant correlation towards better clinical outcome at 3 months across time with EVT therapy, but not IVT or IAT. There was a statistically significant correlation towards increased recanalization across time with IAT and EVT therapy, but not IVT therapy. There was a statistically significant positive correlation towards decreased SICH across time with EVT therapy, but not IVT or IAT therapy.

Meta-regression demonstrated a statistically significant correlation towards increased mortality across time to groin puncture with IAT and EVT therapy. There was a statistically significant correlation towards lower rates of good clinical outcome at 3 months across time with EVT therapy, but not IAT therapy. There was no statistically significant correlation towards decreased recanalization across time to puncture.

### Heterogeneity statistics and publication bias

See Table 1– Table 4 and Table 7 for heterogeneity statistics and publication bias adjusted weighted pooled rates.

### Discussion

This systematic review and meta-analysis shows better mortality, good clinical outcome and recanalization rates for acute basilar occlusion patients managed with endovascular thrombectomy when compared with either intravenous and/or intraarterial thrombolysis. Further subgroup analysis revealed no significant difference between the use of stent retriever and aspiration thrombectomy. 

### Endovascular thrombectomy versus other approaches

There appears to be no benefit to IV or IA thrombolysis, either alone or in combination, as sole therapy for the management of acute basilar artery occlusion. The results of this meta-analysis show that endovascular thrombectomy is a superior approach to intra-arterial and intravenous thrombolysis, affording statistically significantly lower rates of mortality and symptomatic intracranial haemorrhage, and higher rates of recanalization. The result was also statistically significant for good clinical outcome for endovascular thrombectomy versus intra-arterial thrombolysis, however not versus intravenous thrombolysis. Intra-arterial thrombolysis was not statistically significantly superior to intravenous thrombolysis for both primary and secondary outcomes. However, there are confounding factors that may explain this apparent lack of difference in treatment effect.

These include stroke severity and time to treatment after symptom onset. The BASICS trial ^18^ as one of the studies that held the most weight in the synthesis of outcomes for intravenous thrombolysis. When compared with intra-arterial therapy group, the initial stroke severity was less when comparison is made with baseline NIHSS (21 vs 25). Additionally, time to therapy was also better, with 81% in the IVT group treated in the first 6 hours compared with 64% in the intra-arterial therapy group. This is more marked for the 0-3-hour interval (55% vs 23%). 

Another significant factor is inclusion criteria. Whilst diagnosis of acute basilar artery occlusion and assessment of recanalization is intrinsic to intra-arterial therapy, this is not the case for the IVT group. Most of the patients in the BASICS trial in the IVT group were diagnosed without angiography, and instead with non-invasive modalities such as CTA and MRA. In the study by Lindsberg *et al.* ^6^, the vast majority of patients were included on the basis of MRA (TOF) as opposed to DSA, with MRA (TOF) used to assess recanalization in the days (median 1 day, IQR 1-3) following thrombolysis. This is significant because of potential false positives that may bias the outcome of the study. Other potential confounding factors include thrombus, volume, location and length, and presence of collateral circulation ^2^.

### Factors affecting outcome

The chief aim in the management of patients with acute basilar artery occlusion is the achievement of early recanalization. A meta-analysis by Kumar *et al.* demonstrates that recanalization is associated with a two-fold decrease in death rate (number needed to treat - 2.5) and a 1.5-fold decreased in futile outcome rate (NNT 3) ^5^. However there remains a significant difference between the rate of recanalization and achievement of a good clinical outcome, which is likely due to differences in baseline admission factors.

A meta-regression was performed to assess whether the outcomes of mortality and good clinical outcome systematically varied with time to first groin puncture in the EVT and IAT groups. For the outcome of mortality, there was a statistically significant association for both groups, and for good clinical outcome, there was a statistically significant association for the EVT group. This supports results from the BASICS trial ^18^ and Eckert *et al.* ^19^ which show that the rate of poor outcome was increased when time to recanalization therapy increased, with a significantly higher rate of poor outcomes if the time-period was greater than 6 hours from symptom onset. However, numerous studies in both the IAT and EVT groups find no consistent statistically significant association between time to treatment, and mortality and favourable clinical outcome ^20– 22^. This is likely due to other factors such as collateral flow from posterior communicating arteries and baseline ischemia/infarction.

Baseline ischemia/infarction is another potential factor that affects mortality and clinical outcome. There are two main classification schemes presently, the posterior circulation Acute Stroke Prognosis Early CT Score (pc-ASPECTS) and the brain stem DWI score. Strbian *et al.* showed in a large prospective observation study that the absence of extensive brain infarction is associated with good clinical outcome as measured by the pc-ASPECTS score ^23^. mRS 2-0 at 3 months was seen in 50% of patients with a pc-ASPECTS >= 8 and TIMI 2-3, as opposed to 5.9% in those with pc-ASPECTS <8. Recanalization of up to 48 hours after onset of symptoms was also shown to be beneficial in patients who did not have extensive baseline infarction. Cho *et al.* likewise showed in a cohort of 29 patients treated with intra-arterial therapy that only the brainstem DWI score was associated with futile outcome on both univariate and multivariate analysis ^24^. The implication of this is that a holistic approach is required to determine the optimum treatment approach rather than arbitrary windows of treatment.

Other factors also play a role, and these include age, baseline NIHSS, whether ventilatory support was required, atrial fibrillation, embolic origin and previous stroke ^25^.

### Assessment of heterogeneity

There was significant heterogeneity in the syntheses for the primary outcomes of both the IAT and EVT groups. Sensitivity analyses and meta regression performed helps to explain the heterogeneity found. Meta-regression for the EVT group showed a statistically significant association between the study characteristics of year of publication and time to puncture, and the primary outcome of mortality and mRS 0-2. Meta-regression for the IAT group showed a statistically significant association between the study characteristics of year of publication and time to puncture, and mortality but not mRS 0-2.

IAT studies were very variable in design and ranged across a large span of time from 1986 to 2016 that encompassed significant clinical and technological improvements. Subgroup analysis based on treatment modality showed that the introduction of adjunctive angioplasty +/- stenting resulted in decreased mortality (49.79% vs. 36.55%) and increased rates of good clinical outcomes, (26.48% vs 31.50%), however this was not statistically significant on the z test of interaction (p>0.05), and significant unexplained heterogeneity remains in the synthesis for the primary outcomes.

EVT studies were similarly variable in design with a very heterogenous collection of approaches and devices used. Insignificant heterogeneity was seen for the stent retriever subgroup for the outcomes of mortality, good clinical outcome and SICH and for the thrombo-aspiration subgroup for the outcomes of good clinical outcome, recanalization and SICH. Subgroup analysis however based on treatment modality (SR vs TA) was not statistically significant for any outcome.

Other sources of heterogeneity have the potential to influence the above analyses and these include differences in terms of patient population and size, inclusion and exclusion criteria, definitions, use of adjunctive therapy, follow-up protocol, country of study, and management of relevant physiological parameters such as blood glucose level and blood pressure.

### Endovascular thrombectomy subgroup analysis

There have been significant advances in the mechanical devices used for endovascular thrombectomy, which have resulted in better mortality, good clinical outcome and mortality rates. Broadly, there are two main types of endovascular thrombectomy, the first being stent retriever thrombectomy and the second being aspiration thrombectomy, with combination therapy (Solumbra technique) or switching therapy (e.g. ADAPT) becoming increasingly used.

Weighted pooled analysis of the stent retriever and aspiration thrombectomy subgroups showed no statistically significant difference in the primary and secondary outcomes of mortality, mRS 0-2, recanalization, SICH. There was also no significant different in the safety profile when comparing the outcomes of dissection/perforation and embolization to new territory. However, there was a large imbalance in the number of studies, with significantly fewer analysing the use of aspiration thrombectomy in acute basilar artery occlusion. Recent observational studies by Kang *et al.* ^26^ and Gory *et al.* ^27^ demonstrated similar treatment outcomes between patients who received either stent retriever or aspiration thrombectomy as first-line therapy. However, in the study by Gory *et al.*, thrombo-aspiration was superior in terms of achieving complete perfusion as defined by mTICI 3and shorter length of treatment (0.543 vs 0.315 and 45 vs 56min, p<=0.05 for both) ^27^. This was also seen in studies by Son *et al.* ^28^ and Gerber *et al.* ^29^, but not supported in studies by Kang *et al.* ^26^ and Mokin *et al.* ^30^.

The use of stent retrievers including Solitaire been shown to be superior compared to the earlier retrievers and thrombectomy systems in anterior circulation LVO stroke ^9^. The randomised parallel-group SWIFT trial that compared the Solitaire with the Merci device showed better recanalization, mortality and good clinical outcome rates for the stent retriever device. However, there is only one study by Lutsep *et al.* (n=27) that provides data relating to Merci patients alone, and not in a pooled cohort with other endovascular modalities ^31^. Mortality rate was shown to be 44%, with a recanalization and good clinical outcome rate of 78% and 41%, respectively.

### Limitations

There are numerous limitations to this systematic review and meta-analysis. The main weakness was that data was pooled together from prospective and retrospective observational studies, some including studies with only 10 patients. To date, there has been only one small study with randomised trial data that has looked at therapy pertaining to acute basilar artery occlusion. That particular study only had eight people in each arm (intra-arterial urokinase vs control). In addition, some papers looked more generally at vertebrobasilar or anterior circulation strokes, and in this case, data was separately extracted for outcomes relation to acute basilar artery occlusion or were excluded if this was not possible.

There were also significant differences in terms of patient population and size, inclusion and exclusion criteria, definitions, use of adjunctive therapy, follow-up protocol and time to treatment across the studies. This has resulted in significant heterogeneity in the syntheses for the primary outcomes of both the IAT and EVT group. Sensitivity analyses and meta-regression were conducted to explore this; however, for some analyses, significant unexplained heterogeneity remains. Hence these limitations should be taken into consideration when considering the results of this review.

### Conclusion

In conclusion, the above data supports superior outcomes and better recanalization rates for acute basilar occlusion patients managed with endovascular thrombectomy when compared with either intravenous and/or intraarterial thrombolysis. Further subgroup analysis shows at this stage, there is no significant difference between the use of stent retriever and aspiration thrombectomy both in terms of their efficacy or safety profile. More systematic data is required, preferably randomised clinical trials, to determine the optimal approach to this potentially devastating disease.

### Data availability

### Underlying data

Full reference list for studies included in meta-analysis are available: Open Science Framework: Therapy for acute basilar artery occlusion: a systematic review and meta-analysis, http://doi.org/10.17605/OSF.IO/4A27M ^32^

### Extended data

Supplementary material including forest plots, funnel plots, exclusion sensitivity plots, meta-regression scatter plots and heterogeneity data for primary and secondary outcomes are available: http://doi.org/10.17605/OSF.IO/4A27M ^32^

### Reporting guidelines

PRISMA checklist: http://doi.org/10.17605/OSF.IO/4A27M ^32^
