# Supplementary material for: Therapy for acute basilar artery occlusion: a systematic review and meta-analysis
Source: F1000Res. 2019 Feb 7;8:165. [Version 1] doi: 10.12688/f1000research.18042.1 (PMC6456838; doi:10.12688/f1000research.18042.1)
Supplement: Peer review history

# Peer review: Reviewer response for version 1

**DOI:** 10.5256/f1000research.19731.r45712

**Published:** 2019-04-08

**Authors:** Fana Alemseged

**Affiliations:** 1 Department of Medicine and Neurology, Royal Melbourne Hospital, University of Melbourne, Melbourne, Victoria, Australia

This systematic review and meta-analysis aims to analyse the efficacy and safety of different treatment approaches for acute basilar artery occlusion. The paper reads reasonably well and the authors acknowledged the main limitations of the study. However, the paper could be improved by the following revisions:

1. Abstract: Please add a description of the main outcomes in the methods. Please include relevant definitions for “good clinical outcome” (mRS 0-2), “recanalization” (TICI 2b-3, mTICI 2b-3, TIMI 2-3) and “sICH” (ECASS II).
2. Please cite the results of the BEST trial in the introduction.
3. Please briefly describe the main results in the “Primary outcomes for the IVT, IAT and EVT groups” and “Secondary outcomes for the IVT, IAT and EVT groups”, in addition to the relevant tables and figures.
4. Can the authors provide a descriptive figure for clinical outcome, recanalization and safety profile for ADAPT vs stent retriever thrombectomy?
5. Figures 2b-c and 3b-c are not legible. Please improve the quality of these figures.
6. Please add a comment and cite the following article in the discussion: “ *Firstline a direct aspiration first pass technique versus firstline stent retriever for acute basilar artery occlusion: a systematic review and meta-analysis*” by Ye *et al. *(2019 ^1^).
7. Please rephrase the sentence "There was a statistically significant correlation towards increased recanalization across time with IAT and EVT therapy, but not IVT therapy" for greater clarity.
8. Consider deleting the paragraph on the use of Solitaire vs Merci device in anterior LVO strokes as not pertinent.
9. There are several typos in the abstract, main body of the manuscript and figures: i.e. modified Ranking Score, incleded, etc. Please review the text for accuracy.
10. Please use scientific terminology rather than common language throughout the paper (i.e. people>patients, in different ways>different clinical presentation, etc.). 

I have read this submission. I believe that I have an appropriate level of expertise to confirm that it is of an acceptable scientific standard.

## References

[1] YeGLuJQiPYinXWangLWangD: Firstline a direct aspiration first pass technique versus firstline stent retriever for acute basilar artery occlusion: a systematic review and meta-analysis. *J Neurointerv Surg*.2019; 10.1136/neurintsurg-2018-014573 10.1136/neurintsurg-2018-014573 30692214

# Peer review: Reviewer response for version 1

**DOI:** 10.5256/f1000research.19731.r44183

**Published:** 2019-02-14

**Authors:** José Biller, Ravi Garg

**Affiliations:** 1 Department of Neurology, Stritch School of Medicine, Loyola University Chicago, Maywood, IL, USA

**“Therapy for acute basilar artery occlusion: a systematic review and meta-analysis”**

**Background:**

*Basilar artery stroke is most commonly caused by atherothrombosis and cardio embolism. The clinical manifestations of basilar artery occlusive disease vary according to the site and nature of vascular compromise. Pertinent clinical profiles pertaining to the topic and the discussion include: 1) proximal and middle basilar artery occlusive disease, frequently of atherosclerotic origin accounting for unilateral or bilateral pontine dysfunction, and less often cerebellar, midbrain, occipital, or mesial temporal lobe ischemia; 2) distal basilar artery occlusion ("top of the basilar syndrome"), frequently of embolic origin and accounting for signs of midbrain and thalamic ischemia, occipital and mesial temporal lobe ischemia, or both.*

 The authors performed a systematic review and meta-analysis comparing three methods of recanalization for acute basilar artery occlusion. The authors should be commended for tackling a difficult topic.

**Questions and Comments:**

Title:

- Identified as a systematic review and meta-analysis.

Abstract:

- Consider adding one-two sentences about background/significance/context of basilar artery occlusion. The purpose and objective is made clear. Would clarify what databases are used, study selection criteria, interventions compared, data extraction method (i.e. PRISMA) all in the methodology portion of the abstract. Results are satisfactory. In the limitations section, consider adding a comment about heterogeneity. This is well described in the discussion, but would add something in the abstract.

 

Introduction:

- Describes the importance that the meta-analyses adds to the current literature – no comments.
- Natural History of Disease: No comments.

 

Methodology:

- Search Strategy: Why was only PubMed used? We would recommend cross referencing other data-bases (SCOPUS, Google Scholar, COCHRANE). Were non-English studies included? We would recommend making an explicit statement regarding this detail.
- Data extraction and Statistical Analyses: No comments. Excellent detail.

 

Results:

- We appreciate that results are not in both tabular and text form.

 

Discussion:

- Endovascular thrombectomy versus other approaches: We would expand as much as possible on the last sentence – confounding by virtue of “thrombus volume, location and length, and presence of collateral circulation. This is a **majo**r source of confounding when comparing a thrombolysis responder versus non-responder.
- Consider the following references: Mehta *et al.,* (2012 ^1^), Alemseged *et al.,* (2017 ^2^) and Goyal *et al.,* (2016 ^3^).
- In the above meta-analyses by Goyal *et al.* (2016 ^3^), consider commenting on the Forest Plot in Figure 2. No subgroup effect of alteplase, but **also **no evidence of effect modification. This supports your paper’s conclusion.
- Would re-search the literature for updates in this arena, as it would greatly add biological plausibility to your clinical conclusion.
- Factors affecting outcome: No comments
- Assessment of heterogeneity: No comments.
- Limitations: No comments.

 

 Overall, this is an excellent paper. We suggest from a methodology standpoint to stay as close to PRISMA guidelines as possible. Additionally, we suggest expanding on sources of confounding when comparing recanalization therapies.

 José Biller, MD, FACP, FAAN, FANA, FAHA

 Ravi Garg, MD

We have read this submission. We believe that we have an appropriate level of expertise to confirm that it is of an acceptable scientific standard.

## References

[1] MehtaBPNogueiraRG: Should clot composition affect choice of endovascular therapy?. *Neurology*.2012;79(13 Suppl 1) : 10.1212/WNL.0b013e3182695859 S63-7 10.1212/WNL.0b013e3182695859 23008415

[2] AlemsegedFShahDGDiomediMSallustioFBivardASharmaGMitchellPJDowlingRJBushSYanBCaltagironeCFlorisRParsonsMWLeviCRDavisSMCampbellBC: The Basilar Artery on Computed Tomography Angiography Prognostic Score for Basilar Artery Occlusion. *Stroke*.48(3) : 10.1161/STROKEAHA.116.015492 631-637 10.1161/STROKEAHA.116.015492 28228577

[3] GoyalMMenonBvan ZwamWDippelDMitchellPDemchukADávalosAMajoieCvan der LugtAde MiquelMDonnanGRoosYBonafeAJahanRDienerHvan den BergLLevyEBerkhemerOPereiraVRempelJMillánMDavisSRoyDThorntonJRománLRibóMBeumerDStouchBBrownSCampbellBvan OostenbruggeRSaverJHillMJovinT: Endovascular thrombectomy after large-vessel ischaemic stroke: a meta-analysis of individual patient data from five randomised trials. *The Lancet*.2016;387(10029) : 10.1016/S0140-6736(16)00163-X 1723-1731 10.1016/S0140-6736(16)00163-X 26898852
